# Laparoscopic reconstructive urology

**DOI:** 10.4103/0972-9941.19265

**Published:** 2005-10

**Authors:** Declan Murphy, Ben Challacombe, Abhay Rane

**Affiliations:** Department(s) of Urology, Guy's Hospital, London, UK; * Department(s) of Urology, East Surrey Hospital, UK

**Keywords:** laparoscopy, reconstruction, pyeloplasty, diversion

## Abstract

**Objective:**

Laparoscopic reconstructive urology is undergoing rapid change. We review the current status of laparoscopic reconstructive urology, with particular respect to pyeloplasty and reconstructive ureteric surgery.

**Methods:**

An extensive Medline search of reconstructive laparoscopic procedures was undertaken. The initial reports and large series reports of a range of procedures was examined and summarised. The most commonly practised procedure within this remit is laparoscopic pyeloplasty. Several series of over 100 patients have been published. Success rates average over 90% for laparoscopic pyeloplasty with a low complication rate. Much less common laparoscopic reconstructive urological procedures include ureteric re-implantation, Boari flap, urinary diversion and transuretero-ureterostomy. The results of these are encouraging.

**Conclusions:**

Laparoscopic pyeloplasty may be safely performed by either the transperitoneal or retroperitoneal routes with excellent results. It should be considered the “gold standard” for the management of UPJ obstruction, especially in those patients with significant hydronephrosis, renal impairment or a crossing vessel. Laparoscopic ureteric reimplantation, Boari flap, urinary diversion and transuretero-ureterostomy have been performed by experienced laparoscopic urologists with encouraging results.

## INTRODUCTION

The number and range of laparoscopic reconstructive procedures being undertaken by urologists has increased significantly during the past 15 years. As the skills of urologists have progressed and the technology has evolved, the number of such procedures performed is set to increase. The objective of this review is to evaluate the development and current status of laparoscopic reconstructive urology.

We have conducted an extensive Medline search of reconstructive laparoscopic procedures from 1995 to 2005. The most commonly reported procedures were collated and reviewed. Within this remit, the most commonly performed procedure is laparoscopic pyeloplasty (dismembered and non-dismembered), and this therefore receives most of our attention. We also review urinary diversion, ureteric reimplantation and some less commonly performed procedures. Reconstruction of the urethra following laparoscopic radical prostatectomy is considered in a separate article, as is the reconstructive element of laparoscopic radical cystectomy. The role of robotic systems such as the daVinci™ (Intuitive Surgical, California) master-slave system are not considered.

### Laparoscopic pyeloplasty

Uretero-pelvic junction (UPJ) obstruction is characterised by obstruction to the flow of urine from the renal pelvis to the upper ureter. Hydronephrosis develops as a consequence and progressive renal impairment may ensue if left uncorrected. Primary UPJ obstruction is a congenital condition and may be associated with an aberrant crossing vessel in up to 65% of cases.[1] Patients are frequently diagnosed incidentally by ultrasound imaging, though loin pain, haematuria or urinary tract infection may also be presenting symptoms.

#### Diagnosis:

Intravenous urography or isotope diuretic renography are used to confirm the presence of UPJ obstruction. Combining these modalities allows the degree of hydronephrosis, the presence of a high ureteric insertion, the differential function and the presence of calculi to be ascertained. Contrast CT scanning is useful for detecting aberrant lower pole vessels.

Progressive loss of renal function or the development of complications such as calculi, are imperative indications for intervention, as is ongoing loin pain.

#### Treatment options:

**Figure 1:**
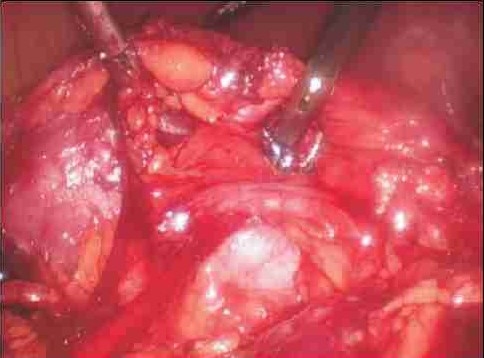
A large lower pole vessel is seen crossing anterior to the right ureter

**Figure 2:**
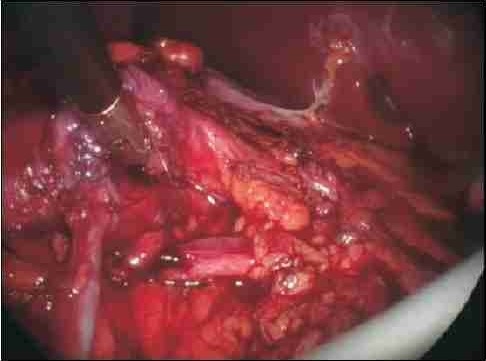
The UPJ (retracted by the laparoscopic instrument) has been repositioned anterior to the crossing vessel

Minimally invasive techniques have been employed in a number of ways for the management of uretero-pelvic junction (UPJ) obstruction. However, techniques such as antegrade endopyelotomy, retrograde endopyelotomy and endoscopic balloon dilatation have proved less effective (56-77% success rate) than open pyeloplasty (>90%) which has remained the “gold standard” after many years of experience. [2–5] Laparoscopic pyeloplasty duplicates the open technique and therefore one would expect similarly high success rates. Pyeloplasty is particularly suitable for patients with a crossing vessel (Figures 1 and 2), severe hydronephrosis, associated calculi or a long stenotic segment.[6] Secondary UPJ obstructions, following failed previous minimally-invasive treatment are also suitable for pyeloplasty.

### History of laparoscopic pyeloplasty

Successful surgical repair of an obstructed UPJ was first described in 1892, one of the earliest true descriptions of a reconstructive urological procedure.[7] The refinement of dismembered pyeloplasty by Anderson and Hynes in 1949 has remained the optimum technique for surgical repair of UPJ obstruction. However the morbidity associated with a flank incision remains significant. Minimally invasive options offer an attractive alternative in this respect.

Laparoscopic pyeloplasty was first reported by Schuessler in 1993.[8] The technique is now in widespread use and several large series have been reported. The success rates for laparoscopic pyeloplasty mirror those of open surgery (89-98% and 86-93% respectively).[3,9,10]

### Laparoscopic pyeloplasty and other minimally-invasive techniques

Though techniques such as percuatneous endopyelotomy, retrograde ureteroscopic endopyelotomy and balloon dilatation have been shown to have a lower success rate than open pyeloplasty, they may still be considered for certain cases. The attraction of lower morbidity, a shorter hospital stay and quicker return to work, have made such techniques more attractive than open pyeloplasty. However patients with crossing vessels, significant hydronephrosis, a long stricture (>2cm), and previous failed endourological treatment, are those most likely to do less well with these minimally invasive techniques.[11] The success rate for percutaneous endopyelotomy in such cases drops from 73% to 39% if the affected kidney has poor function, significant hydronephrosis or a crossing vessel.[12] The presence of a high ureteric insertion on IVU will make an endourological approach less feasible. In these cases, pyeloplasty is much more likely to offer a better outcome.

### Contraindications to laparoscopic pyeloplasty

The presence of a small intrarenal pelvis renders laparoscopic pyeloplasty very difficult and less likely to succeed. Very poor function (< 15%) in the presence of a normal contralateral kidney is an indication for nephrectomy rather than pyeloplasty. Though secondary UPJ obstruction can prove challenging for technical reasons, it is not a contraindication to laparoscopic pyeloplasty. Previous abdominal surgery may prompt one to consider the retroperitoneal rather than transperitoneal approach.

### Laparoscopic *vs* open pyeloplasty

There is no randomised control trial comparing open and laparoscopic pyeloplasty. In a comparative study, Bauer reviewed the outcome of 42 laparoscopic and 35 open pyeloplasties.[10] Success rates exceeded 90% in both groups with a mean follow up of 58 months (minimum 12 months). The complication rate for the laparoscopic group was 12% while that for the open group was 11%. Factors such as hospital stay and recovery times were not considered.

### Operative technique

The choice between the transperitoneal and retroperitoneal approach depends entirely on the surgeon's experience and preference. The precise technical details are not described here. The retroperitoneal space may offer less room to manoeuvre and may make suturing somewhat more challenging, however less dissection is required to access the UPJ and therefore the advantages of one approach usually balance the disadvantages of the other. The retroperitoneal approach may offer shorter operative times (175 vs 246 mins), and there is certainly less risk of intraperitoneal injury and ileus.[13] Our preference is to use this approach using the balloon dissection technique. Four ports are usually necessary to provide the necessary retraction during suturing. A JJ stent is usually placed at the start of the procedure following a retrograde study, however a stent may also be placed antegradely under laparoscopic control if a retrograde study is not necessary prior to insufflation.

### Dismembered *vs* non-dismembered

The majority of authors favour a dismembered technique (Anderson-Hynes), though a non-dismembered approach may be considered in cases without a crossing vessel or significant hydronephrosis. This approach allows excision of a large redundant pelvis, transposition of the UPJ anterior to a crossing vessel, excision of a large stenotic segment, and reconstruction of a dependent non-obstructed UPJ. It also allows the extraction of calculi from the pelvicalyceal system (present in up to 20% of cases). Therefore this technique duplicates that of open dismembered pyeloplasty, which remains the “gold standard” for the treatment of such cases. However such reconstructive surgery is quite challenging laparoscopically. Very good suturing skills must be developed to overcome the learning curve. Operating times average 3 hours for the first 20 cases or so (Table 1).

**Table 1:** Summary of large reported series of laparoscopic pyeloplasty

Series	Number of procedures	Approach (Trans/Retro)	Operating time (mins)	Hospital stay (days)	Success rate (%)	Complications
Hemal[13]	24	Trans 12 Retro 12	187170	4.33.4	95	4% conversion 12.5% ileus
Sundaram [14]	36	Trans	372	2.9	83	3% conversion 11% urine leak
Klingler [17]	40	Trans	N/A	N/A	87.5	2.5% urine leak 5% re-operated 2.5% stricture
Turk[6]	49	Trans	165	3.7	98	2% urine leak
Eden [15]	50	Retro	164	2.6	98	4% conversion
Soulie [9]	55	Retro	185	4.5	87	5.4% conversion 1.8% urine leak 3.6% stricture
Chen [18]	57	Trans	255			
Janetschek [19]	67	Trans & Retro	119	4.1	98.5	1.5% conversion
Jarrett [20]	100	Trans	252	3.3	96	2% urine leak
Inagaki [21]	147	Trans	246	N/A	95	1.5% bowel injury

Of the non-dismembered techniques, a Fenger plasty is most simple. It is suitable for short strictures with minimal hydronephrosis and a low ureteric insertion. A longitudinal incision through the stenotic UPJ is closed transversely in a Heinke-Mikulicz fashion. This obviously allows a much shorter operating time, though one could argue that such cases may also be suitable for one of the endourological approaches referred to earlier.

The other non-dismembered technique that may be considered is the Foley Y-V plasty. The development of a wide-based V-shaped flap of renal pelvis may overcome the difficulties associated with a slightly longer stenotic segment or a high ureteral insertion. It is not suitable for cases with a crossing vessel.

Janetschek et al have reported a success rate of 98% in their series of non-dismembered laparoscopic pyeloplasties. They reserved the dismembered technique for cases with significant hydronephrosis or a high ureteric insertion.

### Complications of laparoscopic pyeloplasty

The commonest complications are bleeding (2%–5.4%), urinary leakage (2%–11%) and stricture formation (2.5%–3.6%). The conversion rate to open surgery is 0%-4% (Table 1).

### Laparoscopic pyeloplasty for secondary UPJ obstruction

Sundaram et al have reported successful treatment of secondary UPJ with laparoscopic techniques.[13] They describe stable or improved renal function in 83% of cases with greater than 50% decrease in pain. Operating times were longer compared to primary UPJ pyeloplasty. Baldwin et al comments that the secondary fibrosis from previous endopyelotomy makes laparoscopic repair more difficult.[3]

### Overall results

The success rate of laparoscopic pyeloplasty equals that of open pyeloplasty. The largest reported series are summarised in (Table 1). The reduction in pain, improvement in function, and improvement in radiographic studies, ranges from 83% to 98%.[14,15] The large series from John Hopkins reports 96% success based on radiological images at a mean of 2.2 years.[16] The incidence of crossing vessels in this series was 56%. The complication rate is low in all large series, with, for example, a stricture rate of 2.5%.[17] However even after more than 50 cases the operating times average over 4 hours in Chen's series.[18] Overall, the three largest series in the literature, offer good evidence that laparoscopic pyeloplasty is a safe and effective procedure. [19–21] The laparoscopic approach also offers lower morbidity, shorter hospital stay and faster return to activity. It is an obvious candidate to become the new “gold standard” for the management of UPJ obstruction. As satisfactory long-term outcome data becomes available this is likely to be confirmed.

### Other laparoscopic reconstructive procedures

*Ureteric re-implantation:* Many of the initial cases of ureteric re-implantation and reconstruction were performed by gynaecologists. They were pioneers in the early days of complex laparoscopy and dealt with any problems as they occurred, laparoscopically if at all possible. One such example is in the management of ureteric obstruction due to endometriosis where Nezhat et al describe three cases of partial ureteric resection and ureteroureterostomy, and one of ureteroneocystostomy.[22] They also describe a total of 9 patients undergoing ureteroureterostomy.[23]

*Laparoscopic boari flap:* Kavoussi's group from Johns Hopkins described the initial cases of laparoscopic Boari flap reconstruction in 2001.[24] They described three patients with ureteric obstruction who were unsuitable for ureteroureterostomy or ureteroneocystostomy because of stricture length and a laparoscopic Boari flap procedure was performed. All procedures were successfully performed without any intra-operative complications or need for open conversion. At roughly the same time Gill's team showed using a porcine model that it was feasible to perform both the refluxing and non-refluxing ureteral re-implantation technique using a Boari flap.[25] The Johns Hopkins group have also used the technique in combination with laparoscopic ureterolysis in the management of retroperitoneal fibrosis.[26]

More recently a Chilean group have described 9 cases of laparoscopic Boari flap.[27] Eight were due to long benign distal ureteric strictures and one was because of transitional cell carcinoma. The mean operative time was 156.6 minutes, mean estimated blood loss 124 cc. There were no intra-operative complications and patients stayed an average of 3 days. Patients were followed up with excretory urograms which looked unobstructed in all cases. While this technique is certainly feasible in the hands of an experienced laparoscopic urologist, it requires advanced suturing skills.

### Retrocaval ureters

Retrocaval ureter is a rare congenital anomaly occurring in one in 1500 people. It is a condition in which the ureter deviates medially and passes behind the inferior vena cava, winding around it and crossing in front of it from medial to lateral side. It was first reported by Hochstetter in 1893 and although the abnormality is congenital, it does not present until the third or fourth decades of life. It commonly presents with right lumbar pain, dull aching or intermittent (renal colic), recurrent urinary tract infections and microscopic or gross haematuria. There is a high incidence of calculi due to stasis and an IVU usually shows an “S” or “fishhook” deformity of the ureter.

The first reports of a laparoscopic treatment for this condition were from Japan: Initially Baba's team from Keio in 1994,[28] followed by Matsuda's group from Osaka,[29] and Ishitoya's group from Kurashiki in 1996.[30] They described successful dissection of the anomalous ureter, division and laparoscopic re-anastomosis (ureteroureterostomy). The first case took a staggering 9 hr, 20 min to complete but operative times are now generally about 2–3 hours. Although the majority of early reports favour the transperitoneal route, this reconstructive procedure is also performed retroperitoneally over a JJ ureteric stent as described by Abbou's group in 1999.[31] It is possible to perform an extra-corporeal anastomosis which may reduce operative time in those not adept at laparoscopic suturing.[32] As with most laparoscopic urological procedures compared with their open counterparts, laparoscopic treatment of retrocaval ureter results in a shorter hospital stay, reduced postoperative pain, early return to daily activities and a superior cosmetic result effect while maintaining functional efficacy.

### Transureteroureterostomy (TUU)

TUU is a urinary reconstruction technique that joins one ureter to the other across the midline. It is used in cases of distal ureteric obstruction due to benign or malignant disease for instance trauma, pelvic malignancies, vesicoureteral reflux, exstrophy, and rare conditions such as amyloidosis which involve large segments of ureter. Generally the accepting ureter must have unobstructed drainage and must not be affected by any disease process that will put both kidneys at risk postoperatively. It is seldom is used if ureteral reimplantation using the psoas hitch or Boari flap is possible.

Laparoscopic feasibility was initially demonstrated in pigs with 8 out of 9 procedures successfully completed and the failure due to an anastomotic stricture in one animal.[33] Ureteroureterostomy in general has also been examined in the porcine model using non-perforating titanium vascular closure staple (VCS) clips in six animals.[34]

### Urinary diversion

Urinary diversion is indicated when the bladder can no longer safely or physiologically function as a reservoir for urine storage. Laparoscopic cystectomy is covered elsewhere so we will concentrate on urinary diversion without cystectomy in the section. The first description of the technique of a Bricker-type laparoscopic urinary diversion was in 1992.[35] The same group from Malaga, Spain reported a clinical case laparoscopic ileal-loop conduit for an elderly high-risk patient with bladder cancer using a four-port technique[36] taking four hours. This group used the extracorporeal anastomosis which is currently employed for many laparoscopic radical cystectomies with ileal conduit formation. In their landmark paper from 1995, they combined the procedure of urinary diversion and cystectomy for the first time.[37] At a similar time Puppo and colleagues reported five cases of urinary diversion via cutaneous ureterostomy,^38^ but once extracorporeal ileal conduit urinary diversion became an established technique it was soon the predominant method employed.

## CONCLUSIONS

Laparoscopic reconstructive urology has evolved rapidly in the past 12 years and will continue to do so. The greatest experience has been gained in laparoscopic pyeloplasty and the many large series published have demonstrated the safety and efficacy of this procedure in the management of UPJ obstruction. As such, the laparoscopic approach should be considered the “gold standard” for the management of such patients. The choice between the transperitoneal or retroperitoneal approach is quite subjective and depends on the experience and preference of the individual surgeon.

Urologists with advanced laparoscopic skills are becoming very adventurous and imaginative in the range of cases they will now consider.[39,40] However not all such reconstructive laparoscopic procedures have achieved full acceptance yet. The complexity and technical skill involved in procedures such as urinary diversion, ureteric reconstruction and retrocaval ureteric surgery will consign such cases to centres of excellence for the immediate future. It is likely that other complex reconstructive procedures will be added to the current list as the skills and experience of laparoscopic urologists develops.

## References

[1] Sampaio FJ (2000). Renal anatomy: endourological considerations. Urol Clin North Am.

[2] Gill HS, Liao JC (1998). Pelviureteric junction obstruction treated with Acucise retrograde endopyelotomy. Br J Urol.

[3] Baldwin DD, Dunbar JA, Wells N (2003). Single-center comparison of laparoscopic pyeloplasty, Accusice endopyelotomy, and open pyeloplasty. J Endourol.

[4] O'Reilly PH, Brooman PJ, Mak S (2001). The long-term results of Anderson-Hynes pyeloplasty. BJU Int.

[5] Notely RG, Beaugie JM (1973). The long-term follow-up of Anderson-Hynes pyeloplasty for hydronephrosis. Br J Urol.

[6] Turk IA, Davis JW, Winkelmann B (2002). Laparoscopic dismembered pyeloplasty - the method of choice in the presence of an enlarged renal pelvis and crossing vessels. Eur Urol.

[7] Kletscher BA, Segura JW, Leroy AJ, Patterson DE (1995). Percutaneous antegrade endoscopic pyelotomy: review of 50 cases. J Urol.

[8] Schuessler WW, Grune MT, Tecuanhuey LV, Preminger GM (1993). Laparoscopic dismembered pyeloplasty. J Urol.

[9] Soulie M, Salomen L, Patard JJ, Mouly PA, Manunta AN, Antiphon PA (2001). Extraperitoneal laparoscopic pyeloplasty: a multicenter study of 55 procedures. J Urol.

[10] Bauer JJ, Bishoff JT, Moore RG, Chen RN, Iverson AJ, Kavoussi LR (1999). Laparoscopic versus open pyeloplasty: assessment of objective and subjective outcome. J Urol.

[11] Tan BJ, Smith AD (2004). Ureteropelvic junction repair: when, how, what?. Curr Op Urol.

[12] Van Cangh PJ, Wilmart JF, Opsomer RJ, Abi-Aad A, Wese FX, Lorge F (1994). Long-term results and late recurrence after endoureteropyelotomy: a critical analysis of prognostic factors. J Urol.

[13] Hemal AK, Goel R, Goel A (2003). Cost-effective laparoscopic pyeloplasty: single center experience. Int J Urol.

[14] Sundaram CP, Grubb RL, Rehman JA, Yan YA, Chen CA, Landman JA (2003). Laparoscopic pyeloplasty for secondary ureteropelvic junction obstruction. J Urol.

[15] Eden CG, Cahill D, Allen JD (2001). Laparoscopic dismembered pyeloplasty: 50 consecutive cases. BJU Int.

[16] Jarrett TW, Chan DY, Charambura TC, Fugita O, Kavoussi LR (2002). Laparoscopic pyeloplasty: the first 100 cases. J Urol.

[17] Klingler HC, Remzi M, Janetschek G, Kratiz C, Marberger MJ (2003). Comparison of open versus laparoscopic pyeloplasty techniques in treatment of uretero-pelvic junction obstruction. Eur Urol.

[18] Chen RN, Moore RG, Kavoussi LR (1998). Laparoscopic pyeloplasty; indications, technique, and long-term outcome. Urol Clin North Am.

[19] Janeteschek G, Frauscher F, Frauscher F (2000). Laparoscopic pyeloplasty. Urol Clin North Am.

[20] Jarrett TW, Chan DY, Charambura TC, Fugita O, Kavoussi LR (2002). Laparoscopic pyeloplasty: the first 100 cases. J Urol.

[21] Inagaki T, Rha KH, Ong AM, Kavoussi LR, Jarrett TW (2005). Laparoscopic pyeloplasty: current status. BJU Int.

[22] Nezhat C, Nezhat F, Nezhat CH, Nasserbakht F, Rosati M, Seidman DS (1996). Urinary tract endometriosis treated by laparoscopy. Fertil Steril.

[23] Nezhat CH, Nezhat F, Seidman D, Nezhat C (1998). Laparoscopic ureteroureterostomy: a prospective follow-up of 9 patients. Prim Care Update Ob Gyns.

[24] Fugita OE, Dinlenc C, Kavoussi L (2001). The laparoscopic Boari flap J Urol.

[25] Fergany A, Gill IS, Abdel-Samee A, Kaouk J, Meraney A, Sung G (2001). Laparoscopic bladder flap ureteral reimplantation: survival porcine study. J Urol.

[26] Fugita OE, Jarrett TW, Kavoussi P, Kavoussi LR (2002). Laparoscopic treatment of retroperitoneal fibrosis. J Endourol.

[27] Castillo OA, Litvak JP, Kerkebe M, Olivares R, Urena RD (2005). Early experience with the laparoscopic boari flap at a single institution. J Urol.

[28] Baba S, Oya M, Miyahara M, Deguchi N, Tazaki H (1994). Laparoscopic surgical correction of circumcaval ureter. Urology.

[29] Matsuda T, Yasumoto R, Tsujino T (1996). Laparoscopic treatment of a retrocaval ureter. Eur Urol.

[30] Ishitoya S, Okubo K (1996). Arai Y Laparoscopic ureterolysis for retrocaval ureter. Br J Urol.

[31] Salomon L, Hoznek A, Balian C, Gasman D, Chopin DK, Abbou CC (1999). Retroperitoneal laparoscopy of a retrocaval ureter. BJU Int.

[32] Tobias-Machado M, Lasmar MT, Wroclawski ER (2005). Retroperitoneoscopic surgery with extracorporeal uretero-ureteral anastomosis for treating retrocaval ureter. Int Braz J Urol.

[33] Dechet CB, Young MM, Segura JW (1999). Laparoscopic transureteroureterostomy: demonstration of its feasibility in swine. J Endourol.

[34] Maxwell KL, McDougall EM, Shalhav AL, Elbahnasy AM, Hoenig DM, Humphrey PA (1998). Laparoscopic ureteroureterostomy using vascular closure staples in porcine model. J Endourol.

[35] Sanchez de Badajoz E, del Rosal Samaniego JM, Gomez Gamez A, Burgos Rodriguez R, Vara Thorbeck C (1992). Laparoscopic ileal conduit. Arch Esp Urol.

[36] Vara-Thorbeck C, Sanchez-de-Badajoz E (1994). Laparoscopic ileal-loop conduit. Surg Endosc.

[37] Sanchez de Badajoz E, Gallego Perales JL, Reche Rosado A, Gutierrez de la Cruz JM, Jimenez Garrido A (1995). Laparoscopic cystectomy and ileal conduit: case report. J Endourol.

[38] Puppo P, Perachino M, Ricciotti G, Bozzo W (1994). Laparoscopic bilateral cutaneous ureterostomy for palliation of ureteral obstruction caused by advanced pelvic cancer. J Endourol.

[39] Kaouk JH, Gill IS (203). Laparoscopic reconstructive urology. J Urol.

[40] Tegavarupu SP, Dasgupta P (2005). Laparoscopic reconstructive urology. BJU Int.

